# Hybrid kernelised expectation maximisation for Bremsstrahlung SPECT reconstruction in SIRT with ^90^Y micro-spheres

**DOI:** 10.1186/s40658-022-00452-4

**Published:** 2022-04-04

**Authors:** Daniel Deidda, Ana M. Denis-Bacelar, Andrew J. Fenwick, Kelley M. Ferreira, Warda Heetun, Brian F. Hutton, Andrew P. Robinson, James Scuffham, Kris Thielemans

**Affiliations:** 1 National Physical Laboratory, Teddington, UK; 2 Institute of Nuclear Medicine, University College London, London, UK; 3 Christie Medical Physics and Engineering (CMPE), The Christie NHS Foundation Trust, Manchester, UK; 4 The University of Manchester, Manchester, UK; 5 Department of Medical Physics, Royal Surrey NHS Foundation Trust, Guildford, UK

**Keywords:** Bremsstrahlung imaging, Tomographic image reconstruction, Synergistic image reconstruction, Kernel method, SPECT-CT

## Abstract

**Background:**

Selective internal radiation therapy with Yttrium-90 microspheres is an effective therapy for liver cancer and liver metastases. Yttrium-90 is mainly a high-energy beta particle emitter. These beta particles emit Bremsstrahlung radiation during their interaction with tissue making post-therapy imaging of the radioactivity distribution feasible. Nevertheless, image quality and quantification is difficult due to the continuous energy spectrum which makes resolution modelling, attenuation and scatter estimation challenging and therefore the dosimetry quantification is inaccurate. As a consequence a reconstruction algorithm able to improve resolution could be beneficial.

**Methods:**

In this study, the hybrid kernelised expectation maximisation (HKEM) is used to improve resolution and contrast and reduce noise, in addition a modified HKEM called frozen HKEM (FHKEM) is investigated to further reduce noise. The iterative part of the FHKEM kernel was frozen at the 72nd sub-iteration. When using ordered subsets algorithms the data is divided in smaller subsets and the smallest algorithm iterative step is called sub-iteration. A NEMA phantom with spherical inserts was used for the optimisation and validation of the algorithm, and data from 5 patients treated with Selective internal radiation therapy were used as proof of clinical relevance of the method.

**Results:**

The results suggest a maximum improvement of 56% for region of interest mean recovery coefficient at fixed coefficient of variation and better identification of the hot volumes in the NEMA phantom. Similar improvements were achieved with patient data, showing 47% mean value improvement over the gold standard used in hospitals.

**Conclusions:**

Such quantitative improvements could facilitate improved dosimetry calculations with SPECT when treating patients with Selective internal radiation therapy, as well as provide a more visible position of the cancerous lesions in the liver.

## Introduction

Selective internal radiation therapy (SIRT) with ^90^Y microspheres, also known as radioembolisation, is a recommended treatment [1–3] for metastatic colorectal cancer and hepatocellular carcinoma. It involves the injection of radioactive microspheres through the hepatic arteries, which flow preferentially to tumours due to their increased vasculature. The microspheres lodge in the blood capillaries of the tumour, delivering a high localised absorbed dose while minimising the dose absorbed by the healthy liver parenchyma [4, 5].

As beta particles emitted by ^90^Y are deflected by atomic nuclei in tissue, Bremsstrahlung photons are produced making imaging following the therapy feasible. Imaging with single photon emission computed tomography (SPECT) is used after the treatment to qualitatively assess the activity distribution of the radionuclide [6–8].

A very small percentage of the emitted gamma photons (32 per million decays) can produce positrons via pair production, making it possible to acquire ^90^Y data with both positron emission tomography (PET) and SPECT. Different studies have investigated the use of PET as a substitute for post-SIRT imaging and provided promising results [4, 9, 10]. This study however focuses on the case where only SPECT acquisition is possible.

There are a number of challenges that degrade image quality and quantification in ^90^Y SPECT images. Firstly, traditional single photon imaging is based on the detection of mono-energetic photons and the images of broad-spectrum Bremsstrahlung photons have degraded spatial resolution (up to 20 mm) and artefacts [11, 12]. In fact, attenuation is estimated assuming mono energetic photons which is not true for ^90^Y given the wide energy spectrum [13]. Scatter correction methods utilising multiple energy windows cannot be used due to the continuous Bremsstrahlung spectrum and there is no peak that can be distinguished from the scattered photons and some studies have attempted the use of monte carlo (MC) techniques [6]. In addition, high energy photons may not be fully attenuated by collimator septa and low energy photons undergo scattering interactions with the collimator. Clinical protocols are based on previous studies which have shown that image quality can be improved by limiting the energy window and using a medium energy collimator. Different studies however have shown inconsistent energy windows and different hospitals may use a different energy window[14–17]. Even though the optimisation of the protocol provides improvements, the partial volume effect (PVE) still makes quantification difficult. Given that all the afore mentioned challenges contribute to the degradation of resolution and the over-smoothing of SPECT lesions, we propose and investigate a solution that reduces PVE and noise by using a synergistic reconstruction taking advantage from anatomical and functional information. The Hybrid Kernelised Expectation Maximisation (HKEM) [18–20] has been demonstrated to improve accuracy and facilitate the identification of small lesions. Such characteristics make this method useful to correctly identify where the micro-spheres deposited. HKEM was derived from KEM proposed by Wang and Qi [21] to improve reconstruction of short frames by using a selection of reconstructed frames as side information, and Hutchcroft et al. [22] where the side information was from magnetic resonance imaging (MRI). In the following years different studies have shown the potential of this technique [23–31] and it has been used in clinical applications such as cardiovascular imaging [32, 33] and cancer theranostics [34].

One potential disadvantage of HKEM is instability in terms of noise propagation of the hybrid kernel over the iterations, which can be further amplified by the lack of scatter correction. We therefore extend the HKEM algorithm to allow the iterative part of the kernel to be frozen at a chosen iteration number so that the kernel is based on an image with lower noise. We refer to this technique as frozen HKEM (FHKEM).

This paper is organised as follows. “Methods” section describes the mathematical aspects of the hybrid kernelized reconstruction algorithm and the frozen extension. It also describes the experimental methodology, reconstruction and the image analysis. “Results and discussion” section presents results and a comparison of the different standard algorithms, and a discussion of these results is provided. In “Further remarks and limitations” section further remarks and the limitations are described and conclusions are drawn in “Conclusion” section.

## Methods

### Algorithm description

In the kernel method, we consider the image, $\lambda$, written as a linear combination

1 $$\begin{aligned} \lambda _j= \sum _{g=1}^{N_j} \alpha _g k_{gj} ; \end{aligned}$$

where $k_{gj}$, is the $gj^{th}$ element of the kernel matrix, *k*, $\alpha _g$ is the $g^{th}$ element of the coefficient vector that we need to estimate, and $N_j$ is the number of feature vectors used to estimate the kernel element relative to the image voxel *j*. Note that the usual additive term used for scatter correction is not present in this formulation since no scatter correction was performed. The FHKEM algorithm can then be described as:

2 $$\begin{aligned} \normalsize \alpha ^{(n+1)}_g = \frac{ \alpha ^{(n)}_g }{\sum _{j=1}^{N_g} k^{(f_{(n)})}_{gj} \sum _{i \in J_g} c_{ij}} \sum _{j=1}^{N_g}k^{(f_{(n)})}_{gj}\sum _{i=1}^{L} c_{ij}\frac{ y_i }{\sum _{l \in I_i} c_{il} \sum _{q=1}^{N_l} k^{(f_{(n)})}_{ql}\alpha ^{(n)}_q }; \end{aligned}$$

where

$$\begin{aligned} {\left\{ \begin{array}{ll} f_{(n)}=n, &{} \hbox {if} f_{(n)}< \hbox {} F_{(n)} \\ f_{(n)}=F_{(n)}, &{} \hbox {if} f_{(n)}\ge \hbox {} F_{(n)} \end{array}\right. } \end{aligned}$$

In the original HKEM, $f_{(n)}$ is the kernel iteration number, which in contrast to *n* will be “freezed”. For the HKEM, $f_{(n)}$ is always equal to the iteration number *n*, while for FHKEM, $f_{(n)}$ stops being updated when it reaches the selected iteration $F_{(n)}$. It is important to note that when ordered subsets (OS) is used, meaning that the algorithm iterates over subsets of the data instead of the full data, $f_{(n)}$ and *n* are actually sub-iterations. A sub-iteration is a single pass through a subsets of the data, therefore *number* *of* *sub*-*iterations* $=$ *number* *of* $iterations\times subsets$.

$\alpha _g^{n}$ is the $g^{th}$ kernel coefficient estimated at iteration *n*, $y_i$ is the $i^{th}$ sinogram bin, *L* is the total number of bins, $c_{ij}$ is the probability that an event occurring in voxel *j* is detected in the $i^{th}$ sinogram bin.

The kernel matrix calculation is described in detail in [18], However a short description is as follows: the $k_{gj}$ element of the kernel can be written as follows:

3 $$\begin{aligned} k^{(f_{(n)})}_{gj} = k_c({\varvec{v}}_g,{\varvec{v}}_j) k_s({\varvec{z}}^{(f_{(n)})}_g,{\varvec{z}}^{(f_{(n)})}_j) ; \end{aligned}$$

where

4 $$\begin{aligned} k_{c} ({\varvec{v}}_g,{\varvec{v}}_j) = \exp {\left( - \frac{\Vert {\varvec{v}}_g-{\varvec{v}}_j \Vert ^2}{2 \sigma _c^2} \right) } \exp {\left( - \frac{ \Vert {\varvec{x}}_g-{\varvec{x}}_j \Vert ^2}{ 2 \sigma _{\mathrm{dc}}^2} \right) }; \end{aligned}$$

is the kernel coming from the CT image and

5 $$\begin{aligned} k_{s} ({\varvec{z}}^{(f_{(n)})}_g,{\varvec{z}}^{(f_{(n)})}_j) = \exp \left( - \frac{\Vert {\varvec{z}}^{(f_{(n)})}_g-{\varvec{z}}^{(f_{(n)})}_j \Vert ^2}{2 \sigma _s^2} \right) \exp \left( - \frac{\Vert {\varvec{x}}_g-{\varvec{x}}_j \Vert ^2}{ {2 \sigma _{\mathrm{ds}}^2}} \right) . \end{aligned}$$

is the part coming from the SPECT iterative update. The quantity ${\varvec{x}}_j$ is the coordinate of the $j^{th}$ voxel, ${\varvec{z}}^{(f_{(n)})}_j$ is the feature vector that is calculated from the $f^{th}$ SPECT update image, and ${\sigma }_c$, ${\sigma }_s$, $\sigma _{\mathrm{ds}}$ and $\sigma _{\mathrm{dc}}$ are scaling parameters for the distances in (3) and (4). ${\sigma }_c$ and ${\sigma }_s$ control the edge preservation from the anatomical image and functional image, $\sigma _{\mathrm{ds}}$ and $\sigma _{\mathrm{dc}}$ control the weight of the neighbouring voxels based on the distance from the central voxel in the neighbourhood, $\sigma _{\mathrm{ds}}$ and $\sigma _{\mathrm{dc}}$ are in this case redundant as the images have the same dimension and voxel size, as a consequence, even though different values can be set, they are treated as one and $\sigma _{\mathrm{ds}}$ = $\sigma _{\mathrm{dc}}$.

### Phantom data

The phantom data were acquired at the National Physical Laboratory (NPL), UK, using the Mediso AnyScan SCP. Reconstruction for this scanner has previously been implemented [35, 36] in the Software for Tomographic Image Reconstruction (STIR) [37]. A NEMA phantom was scanned containing 6 spherical inserts of different volume and the same $^{90}$Y activity concentration. The diameter of each sphere was 10 mm, 13 mm, 17 mm, 22 mm, 28 mm and 37 mm and the activity at the time of scanning was 0.255 ± 0.001 MBq, 0.511 ± 0.003 MBq, 1.19 ± 0.01 MBq, 2.58 ± 0.01 MBq, 5.34 ± 0.03 MBq, 12.58 ± 0.07 MBq and the background was filled with water. The data were acquired for 2 h, with 120 60 s projections. The energy window was set between 75 and 225 keV. A parallel-hole medium energy general purpose (MEGP) collimator was used. The CT image was acquired for attenuation estimation and was used as anatomical image in the HKEM and FHKEM.

### Clinical data

Clinical data were acquired at the Royal Surrey NHS Foundation Trust in Guildford, UK, using the GE Optima 640 SPECT/CT system for 5 patients who were treated with SIRT with $^{90}$Y resin microspheres (SIR-Spheres). The patient data involved in this study were anonymised. The injected activity was in the range between 1.5 and 2.2 GBq and the SPECT acquisitions lasted 40 min, with 120 20 s projections. The energy window was set between 75 and 225 keV. A medium energy general purpose parallel-hole MEGP collimator was used. The CT image was acquired for attenuation estimation and was used as anatomical image in the HKEM and FHKEM.

### Reconstruction setup and analysis

The point spread function (PSF) for the NPL Mediso gamma camera with collimator was obtained with data of multiple linear $^{90}$Y sources at different distances from the detector. A linear fit was used to study the dependence of the sigma of the Gaussian curve on the distance from the detector. Since no PSF measurements were available for the GE Optima used for the patient data a theoretical PSF model was estimated from the collimator properties [38]. The same model was used for the Mediso scanner and compared to the measured model, and results are discussed below.

All the reconstruction algorithms in the study use OS. For optimisation purposes, many reconstructions were carried out with different combinations of the parameters, such as the number of subsets for all algorithms, set to 12, all the sigma parameters for the HKEM (which are described in Table 1), the number of neighbours, and the iteration at which the iterative kernel is frozen for FHKEM. The anatomical image used as side information is a sequentially acquired CT image which was manipulated to create spatial inconsistencies between CT and SPECT. In particular some of the spheres were removed from the image. The data was reconstructed using ordered subsets expectation maximisation (OSEM) with no PSF (OSEM), OSEM with PSF (OSEM-PSF), HKEM and FHKEM. For the patient data the image reconstructed with OSEM, 2 iteration using GE Xeleris was also added into the comparison. All the (F)HKEM results reported in this manuscript are obtained with the use of the same resolution modelling as the OSEM-PSF. The SPECT image size was 128$\times$128$\times$128, while the voxel size was 4$\times$4$\times$4 mm$^3$. The CT image size for the phantom was 512$\times$512$\times$82, while the voxel size was 0.98$\times$0.98$\times$5 mm$^3$. For the patient the CT image size was 512$\times$512$\times$161, while the voxel size was 0.98$\times$0.98$\times$2.5 mm$^3$. The CT images were re-sampled to match the SPECT image properties.

**Table 1:** FHKEM selected parameter values

FHKEM selected parameter values	
Neighbours	5$\times$5$\times$5
Functional edge $\sigma _s$	1
Anatomical edge $\sigma _c$	0.1
Spatial distance $\sigma _{\mathrm{ds}} = \sigma _{\mathrm{dc}}$	3
Kernel frozen at sub-iteration $F_{(n)}$	72

The choice of the parameter settings was based on the results in Fig. 2, that is, the “best” parameter value is chosen so that it provides the highest uptake while suppressing more noise.

**Fig. 1:**
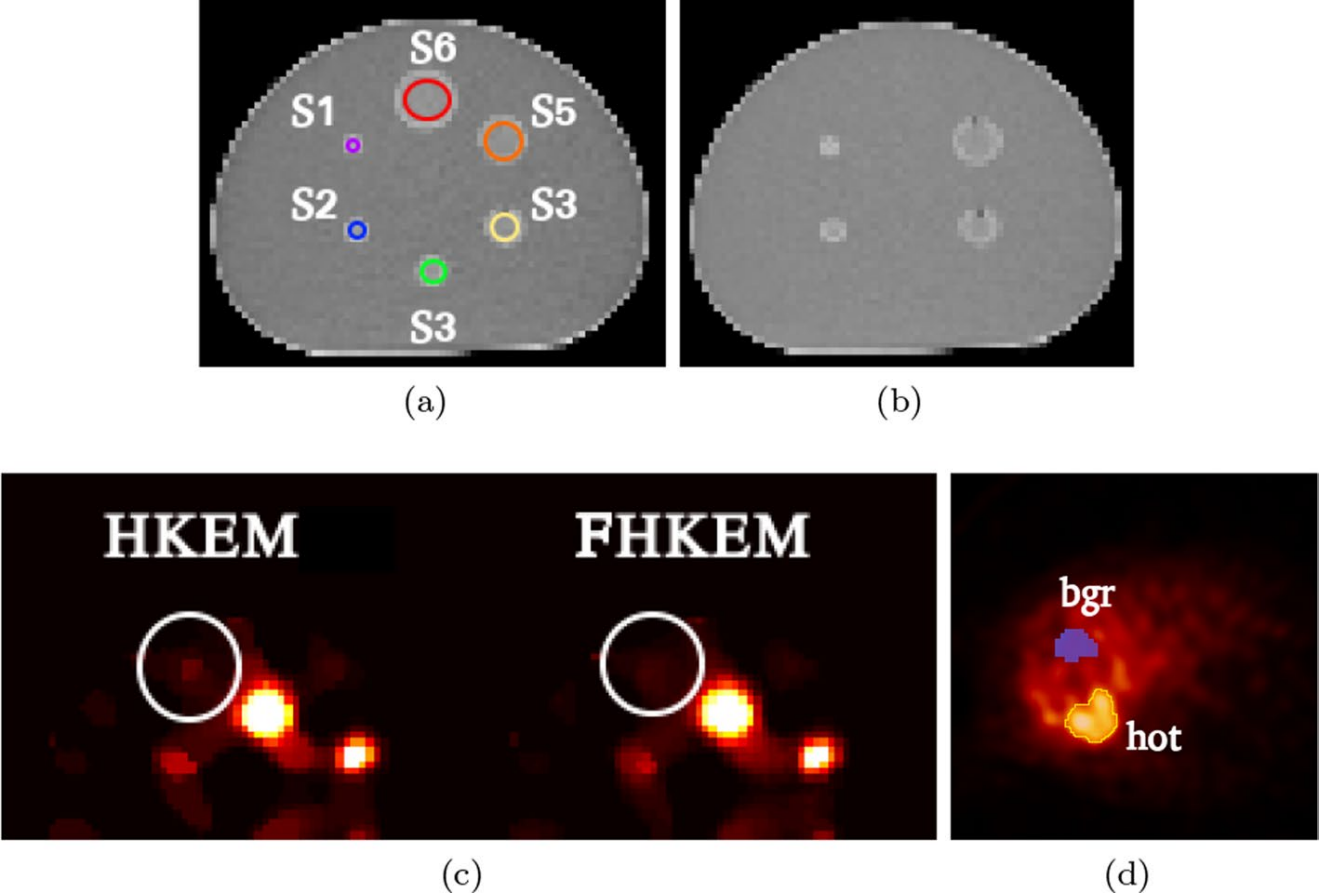
**a** CT image with the ROIs for the NEMA phantom; **b** image used as anatomical image and obtained by removing the spheres S6 and S3 from the CT in 1a; **c** Magnification of the SPECT reconstructed images with HKEM and FHKEM for the NEMA phantom. The image is zoomed in on the phantom area containing spheres S1, S5 and S6. Within the white circle is shown a false small region of activity in the HKEM image which is not shown in FHKEM. **d** Background (bgr) and hot ROIs example for the patient data using FHKEM. All The images show the transaxial direction

To estimate the recovery coefficient, the mean value of the inner voxels in the biggest sphere of the OSEM image was divided by the input activity to determine a “calibration factor”. The ROI used is a sphere with a 3 voxels diameter. In this way we can have a measure of the degradation due to PVE for each sphere. Recovery curve are usually used to correct activity values in patient images, however this is not done here as the patient data is acquired with a different scanner. The ROI analysis was carried out in terms of mean value, CoV, and contrast to noise ratio (CNR) using the ROIs shown in Fig. 1a, d. For the patient data the chosen ROI was extracted from the hottest lesion in the liver and the background (bgr) ROI from the surrounding cold liver.

## Results and discussion

Figure 1c shows a magnification of the SPECT reconstructed images with HKEM and FHKEM for the NEMA phantom. The image is zoomed-in on the phantom area containing spheres S1, S5 and S6. Within the white circle is shown a false small region of activity in the HKEM image which is not shown in FHKEM. If the shape of the phantom was unknown that region could be mistaken for a tumour. In addition, when looking at sphere S1 below the circle, the HKEM image shows an enlargement and distortion of this sphere. To avoid this effects and prevent the noise propagation during the iterative process, we investigated FHKEM.

**Fig. 2:**
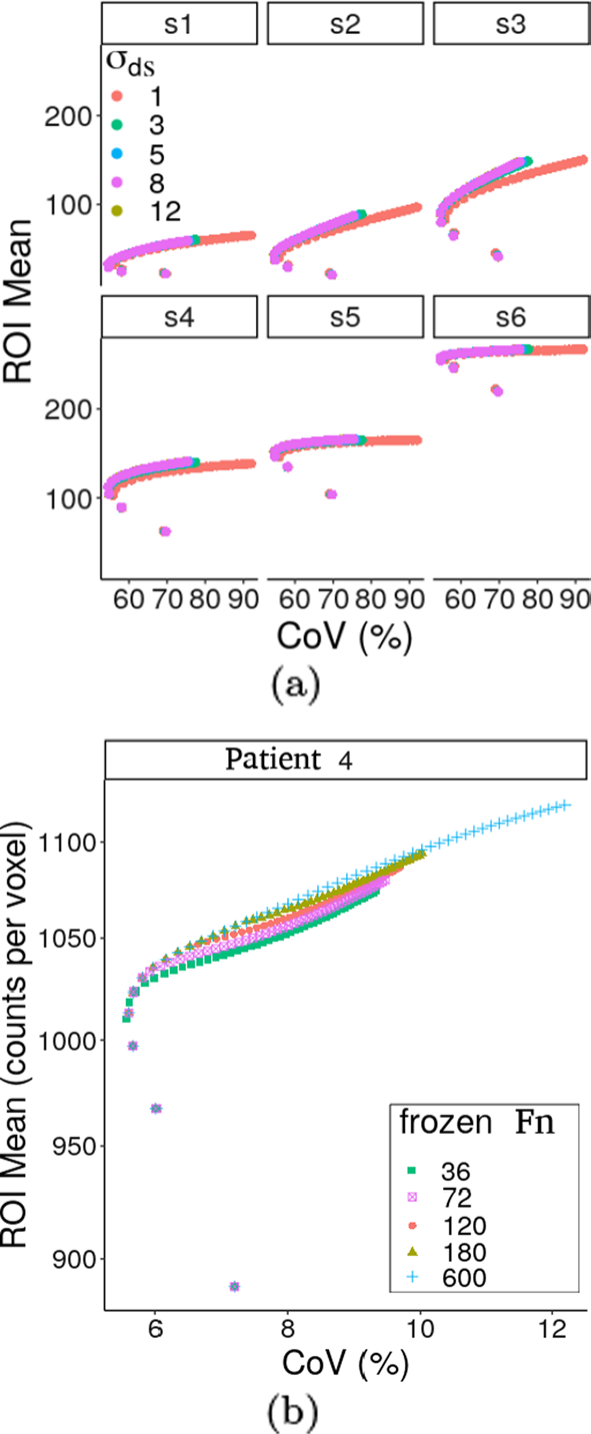
Example of parameter optimisation for the FHKEM. **a** shows the trade-off between ROI mean (kBq/ml) and CoV for the parameter $\sigma _{\mathrm{ds}}$; **b** shows the trade-off between ROI mean and CoV for the iteration where the kernel is frozen, for patient 4

Preliminary investigation on the optimisation of (F)HKEM parameters in terms of ROI values and CoV was carried out. An example of this optimisation is given in Fig. 2a, which reports the trade-off between ROI mean (kBq/ml) and CoV at different values of $\sigma _{\mathrm{ds}}$ while the other parameters are fixed, and Fig. 2b for the choice of the sub-iteration where the kernel is frozen, $F_{(n)}$. In Fig. 2 it is possible to see the criteria of such optimisation, the “best” parameter value is chosen so that it provides the highest uptake while suppressing more noise. For example, it can be seen how $\sigma _{\mathrm{ds}}=1$ voxels provides higher CoV than $\sigma _{\mathrm{ds}} >1$ voxels but without increasing the ROI mean significantly. The same can be seen for $F_{(n)}=600$ (HKEM) and $F_{(n)} <600$ where the relative increase in ROI value is smaller than the relative increase of CoV. In fact, there is a difference of 12% between the CoV obtained with HKEM and FHKEM with $F_{(n)}=72$ while there is only 3% between the mean ROI values. Note that this plot refers to patient 4 as the differences were more visible than in the phantom. The selected parameter values are reported in Table 1.

### Phantom data

**Fig. 3:**
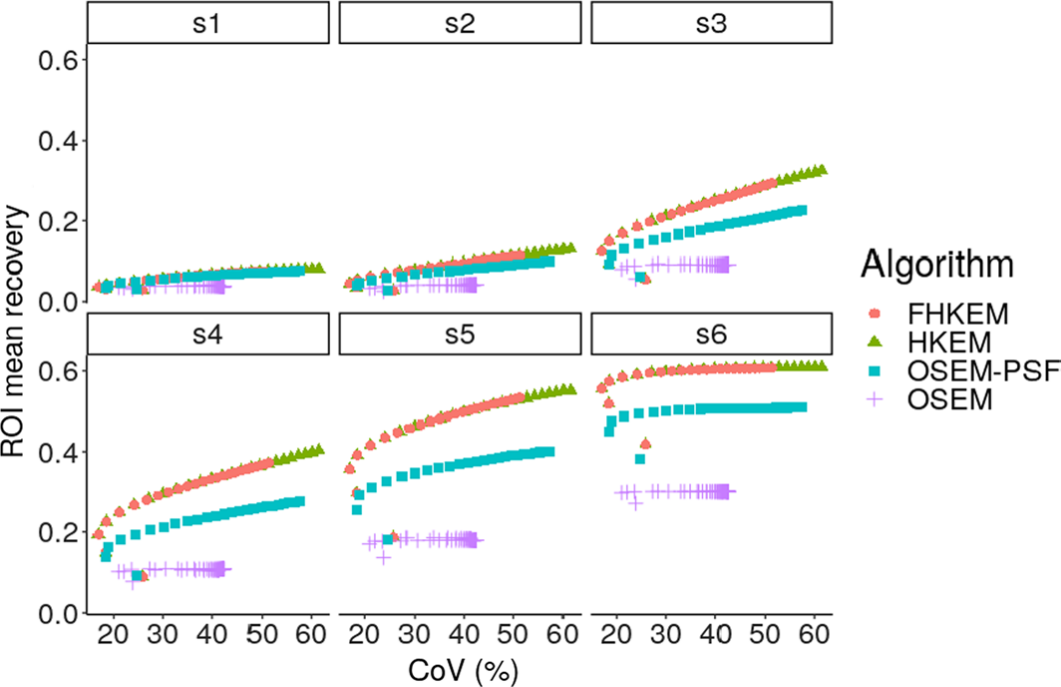
Recovery coefficient comparison between OSEM, OSEM-PSF, HKEM and FHKEM frozen at the 72nd sub-iteration for each sphere

**Fig. 4:**
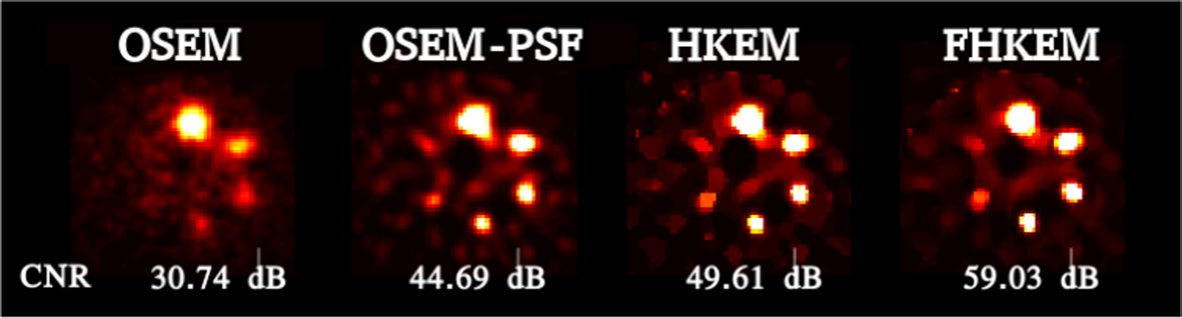
Comparison between reconstructed images of the NEMA phantom using OSEM, OSEM-noPSF, HKEM, and FHKEM with functional kernel frozen at the 72nd sub-iteration. The images correspond to the 50th iteration

**Fig. 5:**
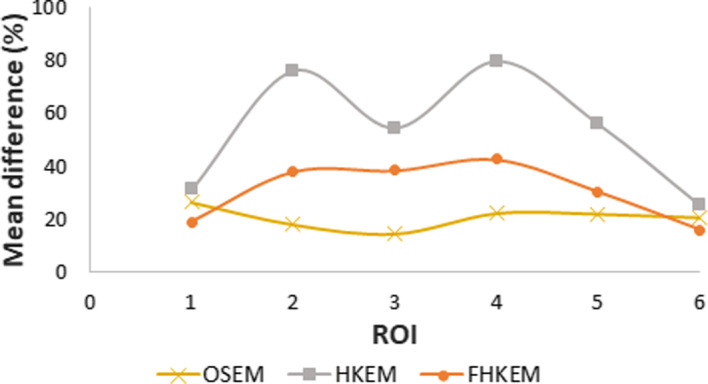
Mean ROI difference between the images reconstructed with the measured PSF (mPSF) model and the estimated PSF (ePSF) model. The plot shows the case of images reconstructed with OSEM, HKEM, and FHKEM. The mean difference is plotted against the ROI number

**Fig. 6:**
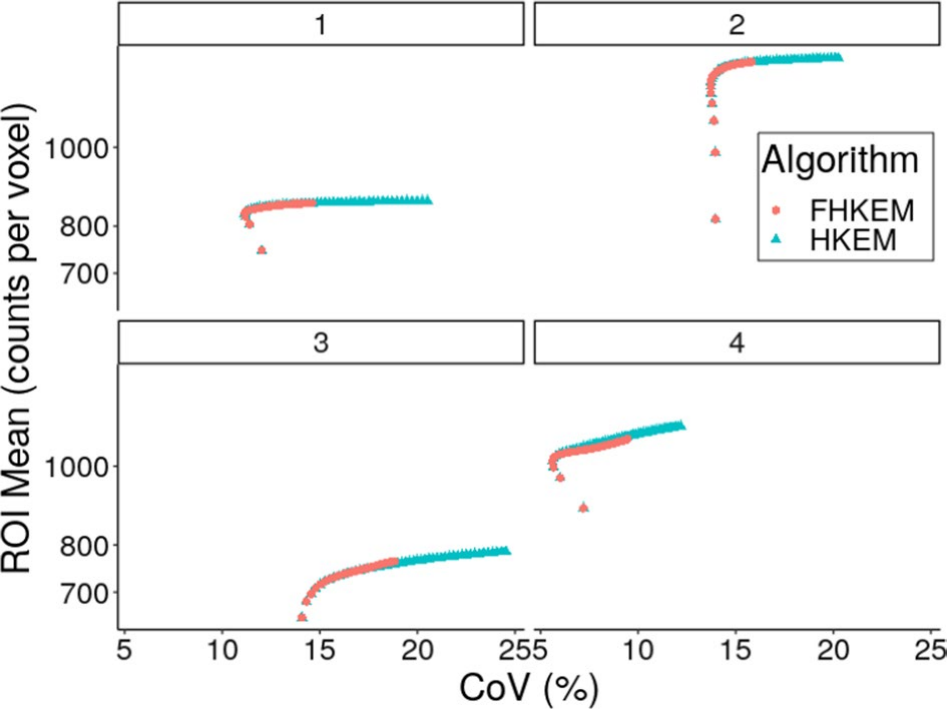
Comparison between HKEM and FHKEM frozen at the 72nd sub-iteration for the hottest lesion of 4 different patients. Comparison of the trade-off between mean ROI value and CoV

**Fig. 7:**
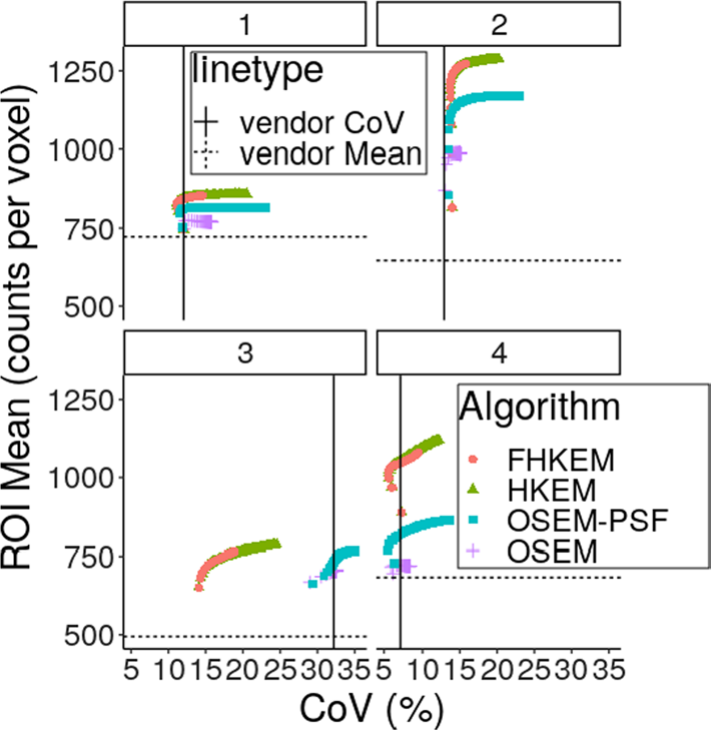
ROI Mean comparison between OSEM, OSEM-PSF, HKEM and FHKEM frozen at the 72nd sub-iteration for the hottest lesion of 4 different patients. The black vertical and horizontal lines represent respectively the CoV and the mean value estimated on the vendor reconstructed image at iteration 2

**Fig. 8:**
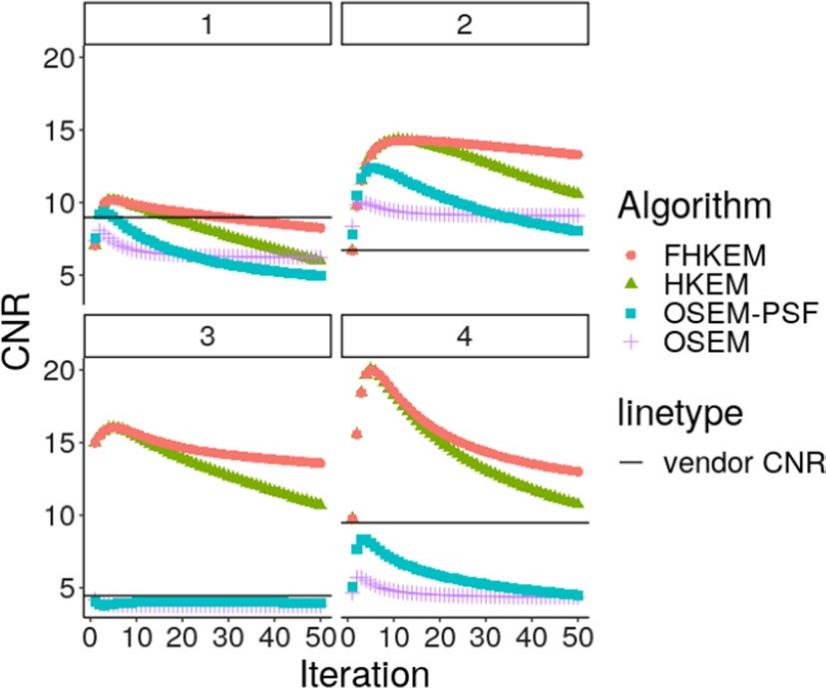
Contrast to noise ratio (CNR) comparison between OSEM, OSEM-PSF, HKEM and FHKEM frozen at the 72nd sub-iteration for the hottest lesion. The black line represent the CNR estimated on the vendor reconstructed image at iteration 2. Legend as per Fig. 7

**Fig. 9:**
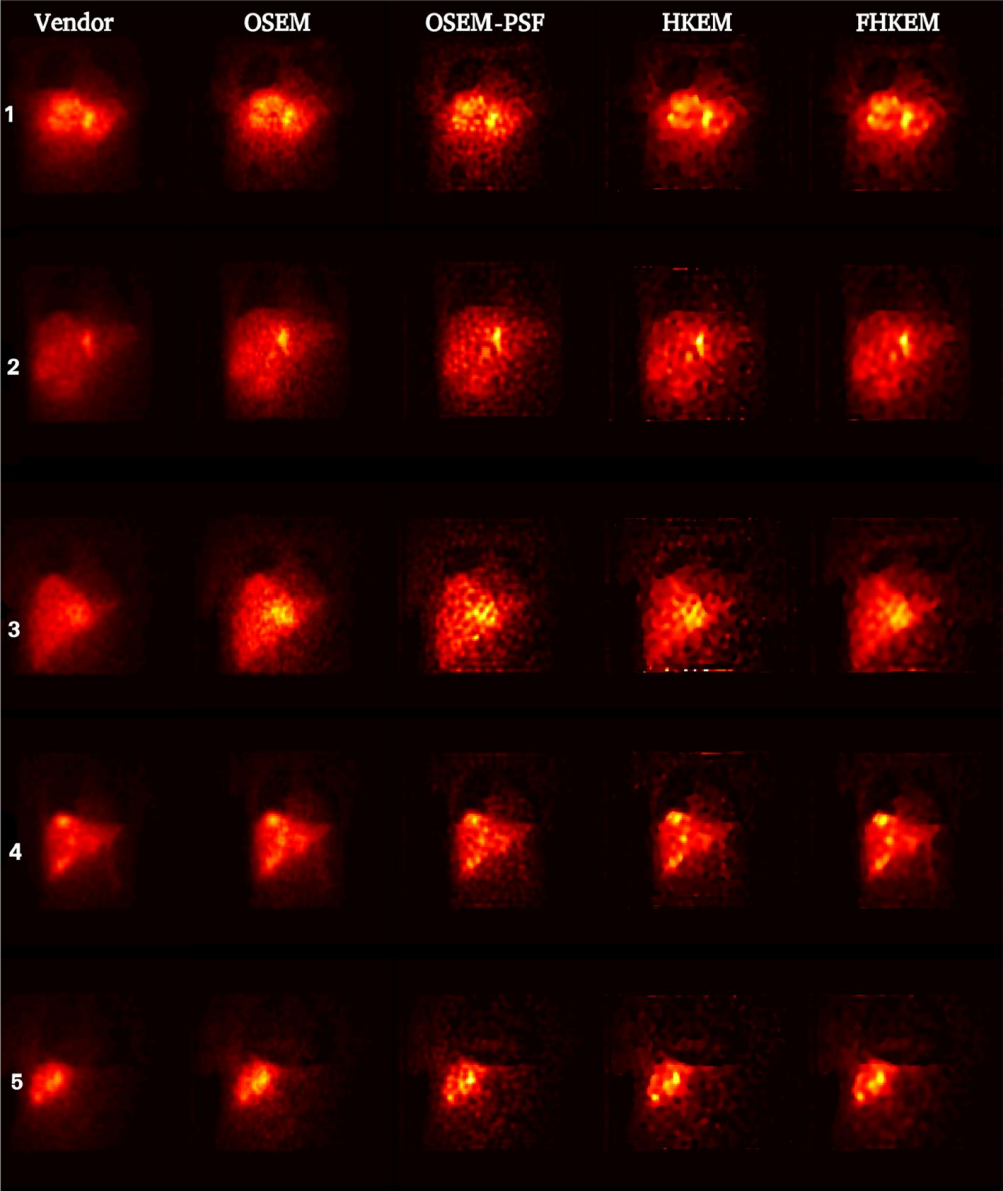
Comparison between reconstructed images of all patients data using the vendor software, OSEM, OSEM-PSF, HKEM and FHKEM with functional kernel frozen at the 72nd sub-iteration. All images use the same scale

**Fig. 10:**
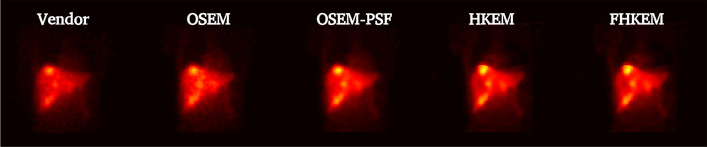
Images at the highest CNR from Patient 4. Comparison between vendor, OSEM, OSEM-PSF, HKEM and FHKEM frozen at the 72nd sub-iteration. All images use the same scale

**Fig. 11:**
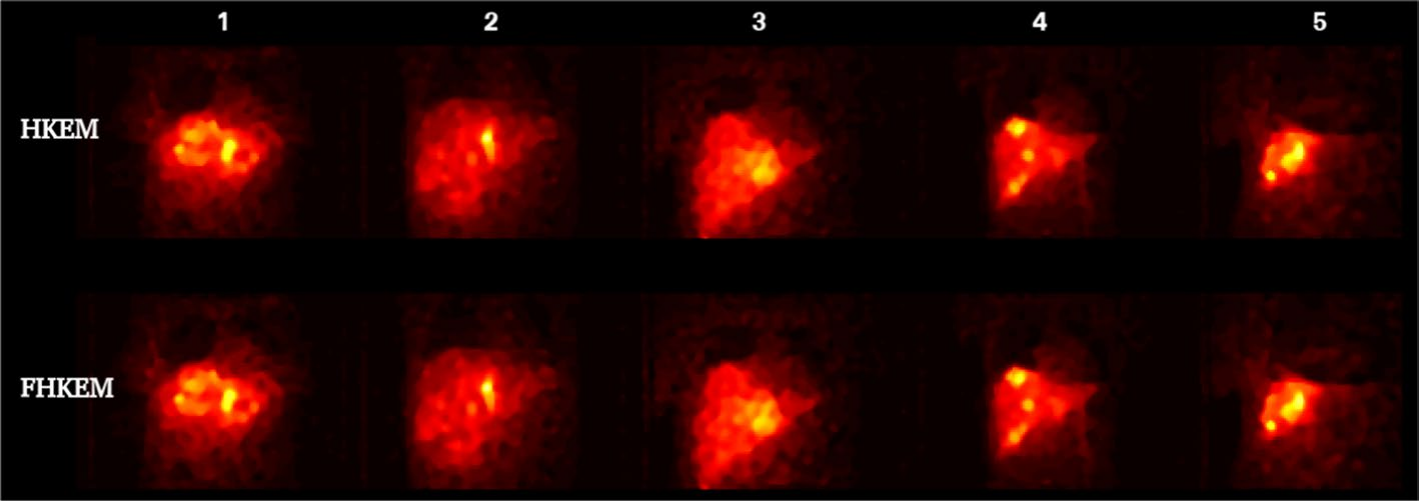
HKEM and FHKEM Images at the optimum Mean-CoV point for all the 5 patients

The comparison between the reconstruction algorithms for the NEMA phantom is shown in Fig. 3, which reports the comparison in terms of the mean value in each ROI versus the CoV in the cold background using OSEM, OSEM-PSF, HKEM and FHKEM with iterative kernel frozen at the 72nd sub-iteration. It can be seen that for all the spheres the PSF provides a big improvement in quantification while the HKEM and  FHKEM outperform the other methods for all regions while providing better noise suppression with a recovery improvement of up to 56% compared to OSEM. The recovery improvement gradually decreases with the size of the lesion. This could be related to the resolution limitation for lesions with size comparable to the kernel neighbourhood. Smaller voxel size could allow further improvement but more investigation is needed. Figure 4, shows the reconstructed images of the NEMA phantom using OSEM, OSEM-PSF, HKEM and FHKEM with functional kernel frozen at the 72nd sub-iteration, (comparison performed at the 50th iteration). A clear improvement can be seen when PSF is used while higher contrast is shown when using HKEM. When looking at the difference between HKEM and FHKEM one can observe that FHKEM can obtain higher contrast spheres, less deformation for the smallest spheres, as well as smoother background.

Since the PSF measurements for clinical scanner was not available, an investigation of the differences obtained when using the measured (mPSF) model and the estimated PSF (ePSF) based on [38] was performed for the phantom data. Figure 5 shows the mean ROI difference between the images reconstructed with OSEM, HKEM and FHKEM using the mPSF model and the ePSF model. The results show that the mPSF is more accurate than the ePSF and that all algorithms show high differences with a maximum of 80% for HKEM, 42% for FHKEM and 27% for OSEM.

### Patient data

When considering the application to patient data Fig. 6 highlights the different behaviour of HKEM when the iterative part of the kernel continues to be updated or it is frozen at the 72nd sub-iteration using the patient data. This shows that while both versions of HKEM reach very close ROI mean values, FHKEM also visibly slows-down the propagation of noise through the iterations.

The comparison between OSEM, OSEM-PSF, HKEM and FHKEM is reported in Fig. 7. In this plot the lesion mean value is plotted against the CoV in the cold liver for 4 different patients. In addition, the vertical and horizontal lines represent the values of ROI mean and CoV for the image reconstructed with the vendor software. The patient data have proven to be consistent with the phantom data. In fact, it can be seen from Fig. 6 that for all the patients the ROI values obtained with HKEM and FHKEM is very close while the CoV becomes more different with higher number of iterations. For example, for patient 3 the improvement in terms of CoV, at iteration 50, of FHKEM over HKEM is 28%. Furthermore, freezing the kernel at early iterations makes the iterative algorithm more stable against noise propagation. Similarly to Figs. 3 and 7 confirms that both HKEM and FHKEM outperform all the other algorithms when it comes to reduce noise while providing activity concentration recovery higher or comparable to the other techniques. In this figure, one can also notice the quantitative improvement over the vendor reconstruction (vertical and horizontal lines). In fact, even considering very early iterations (or similar CoV) the mean ROI value can be improved up to 44% when using HKEM and FHKEM.

In this work the CNR was also considered as a metric of comparison. Figure 8 compares the CNR values obtained using OSEM, OSEM-PSF, HKEM and FHKEM frozen at the 72nd sub-iteration for 4 different patients. The horizontal line indicates the CNR value obtained with the vendor reconstruction. This provides a confirmation of the benefits of HKEM and FHKEM over the other algorithms, and in particular it shows a stabilisation of the CNR for FHKEM due to the “deceleration” of the noise propagation. Note that because the quantitative results for the patients are consistent we are only showing the results for patients 1-4 for style purposes. Nonetheless, to demonstrates the consistency of the results, the images are shown for patient 5 as well.

Finally, Fig. 9 shows the images reconstructed with the vendor, OSEM, OSEM-PSF, HKEM and FHKEM frozen at the 72nd sub-iteration for all patients. The images reconstructed with STIR are all at the 50th full iteration (600 sub-iterations), while the vendor image was reconstructed using 2 full iterations. From a qualitative point of view severe PVE can be seen in the vendor image and OSEM compared to the reconstructions using PSF modelling. The noise, visible in OSEM-PSF has propagated, even though reduced, through HKEM, whereas for FHKEM it is visibly reduced while showing similar contrast.

The CNR figure shows the tendency of the noise to prevail on the contrast when iterating. CNR usually reaches the highest values after a few iterations (5-6 in our case but varies with every patient) in EM algorithms and starts decreasing because of the noise propagation in the iterative process. The ROI mean on the other hand keeps increasing with the iteration and the optimum point needs to be such that the lesion uptake is not severely underestimated. For this reason the optimum iteration for Fig. 6 is different from the one that provides the highest CNR. This can change with the size of the lesion and smaller lesion values take more iterations to converge.

It could be argued that the comparison with the vendor software is not fair as the study uses only a single image (the one used in the clinical examination) at 2 iterations. However, the quantification comparison reports the results of the other algorithms at each iteration. To make the study more complete and fair, in Fig. 10, all reconstructions images are illustrated at the iteration providing the maximum CNR. The figure shows still higher contrast for the FHKEM, however there is no difference between HKEM and FHKEM since the maximum CNR is reached before the kernel is frozen. When looking at the previous plots it can be noticed that at HKEM and FHKEM reach very close optimum points both in terms of ROI mean and CNR and, if we look at the optimum iteration image for both algorithm and all the patients in Fig. 11, it can be seen that the images are very similar. As a consequence, for an application like SIRT, at these energy windows, the HKEM with fewer iterations (10–12) work as well as FHKEM. The benefits of FHKEM are worth in cases where many iterations are needed as it allows convergence stabilisation and avoidance of artifacts at late iterations. FHKEM could play a more important role in cases of lower-count circumstances like a reduction of the energy window. Finally, a reduction of 5% reconstruction time was observed with FHKEM when using a compact kernel matrix which is obtained with feature vectors having only one non-zero elements. But it could make a significant difference when the feature vector contains a higher number of elements, as the HKEM will need to estimate the kernel for every sub-iteration.

### Further remarks and limitations

This study demonstrates qualitative and quantitative improvement for Bremsstrahlung SPECT which could lead to more accurate dosimetry after SIRT with $^{90}$Y.

In light of the results from the PSF investigation it can be asserted that accurate PSF measurements can make a significant quantitative difference. As a consequence, although HKEM and FHKEM already provide improvements over the OSEM, further improvements could be achieved for the clinical data by using a measured PSF.

Further improvement could also be achieved by introducing scatter correction. Several studies have investigated MC simulation techniques for the estimation of the scattered Bremsstrahlung photons [39–42]. However, since a scatter correction means a reduction of counts the FHKEM could have some benefits and in any case the optimum parameter settings for the kernel may need to be tuned.

## Conclusion

This work investigated the use of synergistic reconstruction for Bremsstrahlung SPECT using the HKEM algorithm and an improved version, FHKEM, which enables reconstruction that is more stable against noise propagation. The findings of this study suggest considerable quantitative improvement is possible when using synergistic techniques at lower or comparable noise levels compared to the vendor and STIR OSEM reconstructions. Mean values in hot regions can be improved by up to 56% for phantom data and 47% for the patient data while showing comparable or less noise than the clinical gold standard, OSEM.

## Data Availability

Not applicable.

## Abbreviations

SIRT: Selective internal radiaton therapy
^90^Y: Yttrium-90
HKEM: Hybrid kernelised expectation maximisation
ROI: Region of interest
CoV: Coefficient of variation
CT: Computed tomography
SPECT: Photon emission computed tomography
MC: Monte Carlo
PVE: Partial volume effect
MRI: Magnetic resonance imaging
FHKEM: Frozen hybrid kernelised expectation maximisation
STIR: Software for Tomographic Image Reconstruction
MEGP: Medium energy general purpose
PSF: Point spread function
OSEM: Ordered subsets expectation maximisation
CNR: Contrast to noise ratio
bgr: Background
